# Influence of Ru Content on Defect Formation and Electrocatalytic Activity of Au‐Pd‐Pt‐Ru Compositionally Complex Solid Solution Thin Films for the Hydrogen Evolution Reaction

**DOI:** 10.1002/advs.77600

**Published:** 2026-09-27

**Authors:** Miran Joo, Huixin Xiu, Sabrina Baha, Ridha Zerdoumi, Ningyan Cheng, Christoph Somsen, Yujiao Li, Aleksander Kostka, Wolfgang Schuhmann, Alfred Ludwig, Christina Scheu

**Affiliations:** ^1^ Nanoanalytics and Interfaces Max Planck Institute for Sustainable Materials Düsseldorf Germany; ^2^ School of Materials and Chemistry University of Shanghai for Science and Technology Shanghai People's Republic of China; ^3^ Chair for Materials Discovery and Interfaces Institute for Materials Ruhr University Bochum Bochum Germany; ^4^ Analytical Chemistry‐Center for Electrochemical Sciences (CES) Faculty of Chemistry and Biochemistry Ruhr University Bochum Bochum Germany; ^5^ Chair for Materials Science and Engineering Institute for Materials Ruhr University Bochum Bochum Germany; ^6^ Center for Interface‐Dominated High Performance Materials (ZGH) Ruhr University Bochum Bochum Germany

**Keywords:** compositionally complex solid solution, hydrogen evolution reaction, planar defects, (scanning) transmission electron microscopy, thin films

## Abstract

Compositionally complex solid solutions (CCSSs) consist of a randomly mixed single phase with the potential to enhance electrocatalytic activity through their polyelemental surface atom arrangements. Local structure, chemistry, and lattice strain variation can affect electrocatalytic activity. We investigate the effect of Ru content on defect formation and electrochemistry in Au‐Pd‐Pt‐Ru CCSS thin films. A thin‐film material library covering a wide composition range was fabricated by room‐temperature combinatorial co‐sputtering. High‐throughput characterization, including electron microscopy, X‐ray diffraction, and electrochemical screening, were used to correlate composition and microstructural features with catalytic activity. Three representative compositions – Au_68_Pd_13_Pt_15_Ru_4_, Au_27_Pd_23_Pt_24_Ru_26_, and Au_9_Pd_21_Pt_18_Ru_52_ – were examined. The samples exhibit face‐centered cubic crystal structures, with lattice contraction occurring and a transition from a high density of nanotwins to high‐density stacking faults with increasing Ru content. These planar defects could influence electrocatalytic activity when they terminate at surfaces. Moreover, the hydrogen evolution reaction activity improves with higher Ru content, suggesting that Ru can tune the surface energy for hydrogen adsorption through interactions with neighboring atoms. Atom probe tomography reveals local compositional fluctuations at grain boundaries depending on the Ru content. The findings provide a new insight into surface atom arrangement design in the CCSS electrocatalysts with enhanced performance.

## Graphical abstract

Transmission electron microscopy reveals that the formation of planar defects in Au–Pd–Pt–Ru CCSS thin films depends on Ru content, which in turn influences hydrogen evolution reaction electrocatalytic activity. As the Ru content increases, the density of nanotwins initially rises; with further Ru addition, stacking faults form. These defects may diversify surface atomic arrangements in the CCSS. Overall, these results highlight how microstructural complexity can impact electrocatalytic performance and broaden our understanding of microstructural design strategies for CCSS electrocatalysts.

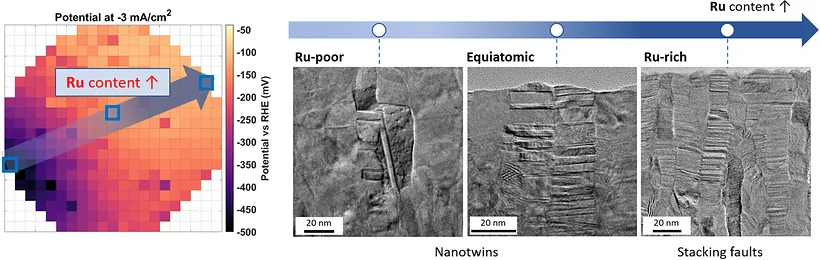

## 1 Introduction

Compositionally complex solid solution (CCSS) systems—consisting of multiple principal elements in a single‐phase solid solution—are of high interest in catalytic materials design [1, 2, 3, 4, 5, 6]. While the demand for efficient catalysts continues to grow, Pt‐based materials remain limited for large‐scale industrial applications due to their high cost and scarcity. To address this, extensive efforts have focused on reducing the Pt content by alloying with transition metals [7, 8, 9, 10, 11]. However, the incorporation of less noble alloying elements often compromises phase stability, undermining the long‐term durability of electrocatalysts and thereby reducing overall electrocatalytic performance [12, 13]. In this regard, CCSS materials offer a promising alternative [14, 15, 16]: by incorporating four or more elements in a highly mixed state, they exhibit high configurational entropy, which helps stabilize the solid solution state during synthesis but could also contribute to maintaining phase stability under electrochemical environments.

The random elemental mixing of CCSS systems gives rise to diverse surface atom arrangements. These arrangements generate a broad distribution of binding energies across the surface, enabling efficient adsorption and desorption of reaction intermediates and ultimately accelerating reaction kinetics [17, 18, 19]. The compositional complexity that provides catalytic versatility can also lead to further properties which could also influence the electrocatalytic activity of these materials. More interestingly, the compositional complexity in CCSS means that the electrocatalytic activity is not only determined by the identity of the active element, but also by the neighboring atoms surrounding the binding site [17], as compared with binary or ternary alloys. In CCSS electrocatalysts for hydrogen evolution reaction (HER), the local atomic arrangement of Au, Pd, Pt, and Ru, can tune the hydrogen binding energy and generate a variety of surface sites with different catalytic activities [20]. In addition, lattice distortions and severe strain accumulation can occur due to the size differences of constituents [21, 22]. To relieve strain, defects such as twins or stacking faults (SFs) can form. While such features may enhance catalytic activity, long‐term stability could pose a critical challenge for CCSS electrocatalysts.

Microstructure introduces additional complexity to CCSS systems. In perfect crystalline solids with long‐range order atoms are periodically arranged. In reality, however, various defects inevitably may form, ranging from point defects such as vacancies to extended defects such as dislocations and grain boundaries (GBs). These features alter local atomic arrangements and electronic structures, thereby also impacting catalytic performance [16]. Moreover, the atom arrangements at surfaces can differ substantially from those in the bulk, especially under electrochemical conditions. Local compositional fluctuations around defect structures are particularly relevant in CCSS systems, where diverse atomic configurations could promote the formation of complex hierarchical microstructures. As a result, defect formation influences catalytic behavior, underscoring the importance of probing structural complexity with advanced characterization methods – a research area that remains underexplored [23, 24, 25, 26].

To date, most investigations of CCSS materials have emphasized microstructural analysis in relation to mechanical properties [27, 28, 29], while relatively limited studies have addressed the atomic‐ and nanoscale structures that directly govern catalytic activity. Although CCSS catalysts have begun to attract attention for electrocatalytic applications, much of the research remains at an early stage focusing largely on exploring preliminary correlations between composition, microstructure and electrochemical behavior. Given the extreme compositional and microstructural complexity of CCSS, detailed characterization is needed to unravel the fundamental physics linking atomic‐scale features to catalytic properties. Because catalytic processes inherently occur at the nano‐ and atomic‐scales, high‐resolution (scanning) transmission electron microscopy (HR‐(S)TEM) techniques, in combination with high‐throughput characterization and other advanced characterization tools are required to select the most relevant samples from the large compositional ranges of interest. This is especially important for investigating the complexity and establishing the interconnections between composition, structure and activity.

This study investigates the effect of the Ru content on the defect formation and electrocatalytic properties in Au‐Pd‐Pt‐Ru CCSS thin films, with a special focus on planar defects in dependence of the increasing Ru content. Our study aims to develop a fundamental understanding of how Ru content and planar defect formation influence the HER electrocatalytic activity, rather than achieving the highest performance. High‐throughput (screening) methods including scanning electron microscopy (SEM) with energy dispersive X‐ray spectroscopy (EDX), X‐ray diffraction (XRD), and scanning droplet cell (SDC) measurements allow us to correlate the average chemical composition and crystal structure with electrocatalytic activity. On a local scale, HR‐(S)TEM analysis reveals grain morphology and composition distribution, and further provides the detailed structural information on nanotwins and SFs that develop in the thin films depending on the Ru content. Additionally, atom probe tomography (APT) highlights local atomic fluctuation around GBs, supporting our understanding of the compositional and microstructural complexity in the CCSS thin films.

## 2 Results and Discussion

**FIGURE 1:**
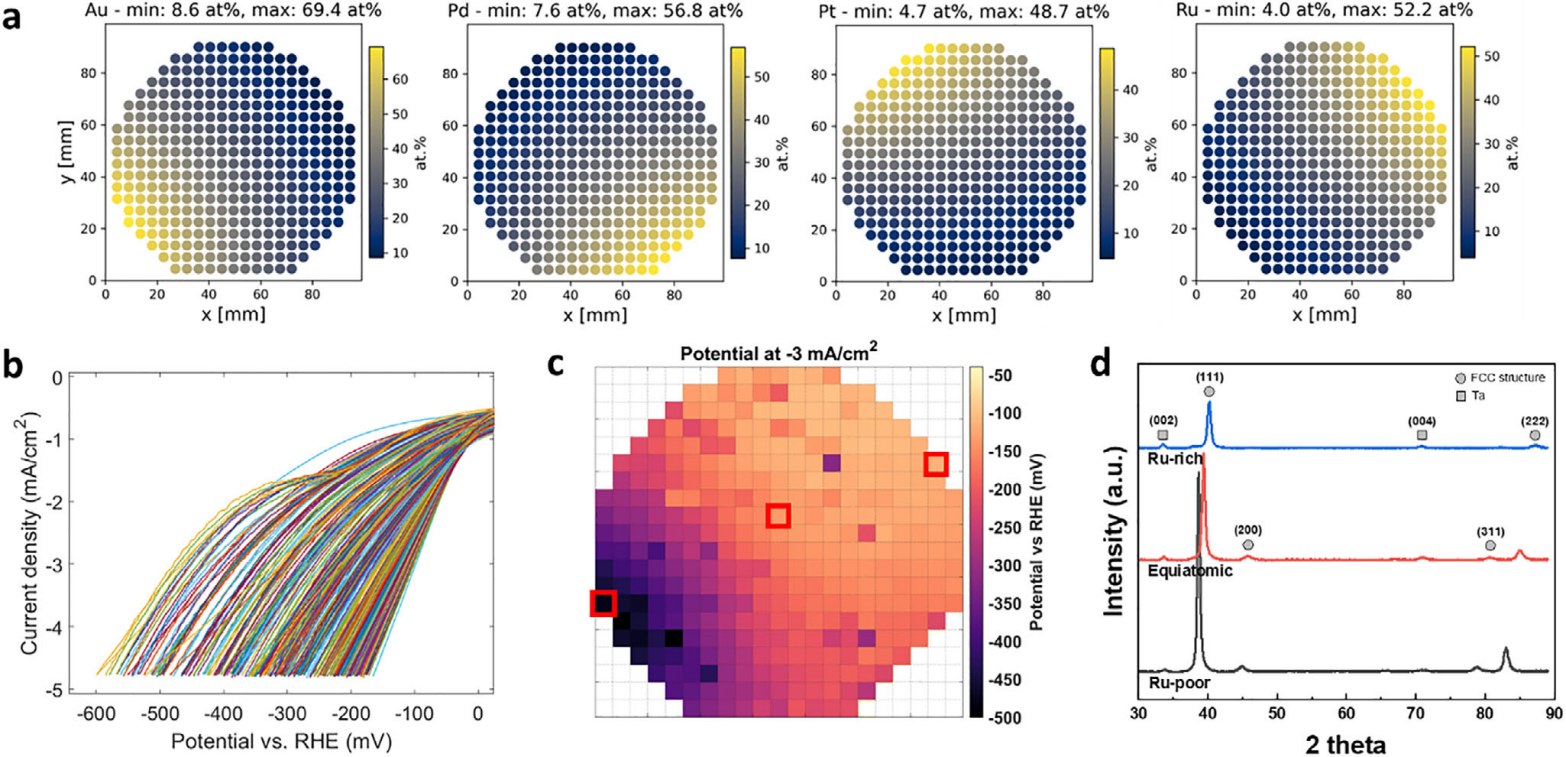
High‐throughput screening data (a) Color‐coded composition maps of Au, Pd, Pt, and Ru in the quaternary Au‐Pd‐Pt‐Ru CCSS thin‐film materials library. (b) Linear sweep voltammograms acquired using a high‐throughput scanning droplet cell in the hydrogen evolution region. (c) Electrocatalytic activity map (overpotential at a current density of −3 mA cm^−2^) with the preselected MAs for further TEM analysis marked in red squares. (d) Corresponding XRD patterns of the three MAs of the thin‐film library.

Figure 1a shows maps of the compositional gradients of the quaternary thin‐film materials library Au–Pd–Pt–Ru system fabricated by magnetron co‐sputtering. Each map spans a wide compositional range, with the elemental contents ranging from low to high across the library, corresponding to the positions of the four pure‐element sputter targets. With the targets positioned at the sputter chamber corners, composition gradients are achieved across the library, with the equiatomic composition located near the center of the sapphire substrate. High‐throughput SEM‐EDX measurements were used to determine compositional distributions across the library. The nominal compositions of three representative measurement areas (MAs) are listed in Table S1. These values agree closely with low‐magnification STEM‐EDX measurements (Table S1), confirming the reliability of the compositional analysis.

The electrocatalytic activity of the materials library for the HER was investigated in 0.05 m KOH using a high‐throughput SDC. The linear sweep voltammograms (LSVs), normalized to the geometric surface area, are shown in Figure 1b. The potential required to reach a current density of −3 mA/cm^2^ was then determined from the LSVs and plotted as an activity map in Figure 1c. The activity map reveals a strong compositional dependence: Ru‐rich regions (upper right) exhibit significantly higher activity (lower overpotential), while Ru‐poor (Au‐rich) regions show a lower activity. The systematic increase in activity with Ru content suggests that higher Ru contents tune the surface energies for hydrogen adsorption by modifying the electronic structure of the active sites with neighboring elements. It is worth noting that the electrochemical measurements for the HER activity were conducted in the presence of atmospheric oxygen, and therefore, the LSVs may contain a contribution from the oxygen reduction reaction (ORR), especially in the low‐current regions. Therefore, selecting a relatively high current density of ‐3 mA/cm^2^, where the Faradaic response is primarily due to the HER, effectively removes the ORR background to a large extent. To establish detailed correlations between composition, structure and activity, three representative compositions (marked with red squares on the activity map) were selected for further investigations: Au_68_Pd_13_Pt_15_Ru_4_ (Ru‐poor), Au_27_Pd_23_Pt_24_Ru_26_ (equiatomic), and Au_9_Pd_21_Pt_18_Ru_52_ (Ru‐rich).

XRD patterns of the three thin films show face‐centered cubic (FCC) crystal structures (solid circles), with no evidence of hexagonal close‐packed (HCP) phases (Figure 1d). A diffraction peak from the Ta adhesion layer (solid square) between the films and the sapphire (0001) substrate is observed. With increasing Ru content, the diffraction peaks shift to higher angles, indicating lattice contraction. Although pure Ru is thermodynamically stable in the HCP phase, it is stabilized in the FCC structure of the quaternary solid solution [30]. Given that FCC Ru (∼0.380 nm) [31] has a smaller lattice parameter than FCC Au (∼0.407 nm), the observed peak shift is consistent with Ru alloying. Lattice parameters, calculated from the (111) and (222) reflections using Bragg's law, are ∼0.403 nm (Ru‐poor), ∼0.395 nm (equiatomic), and ∼0.388 nm (Ru‐rich), consistent with the lattice parameters of the constituent elements. The use of (111) and (222) reflections ensured accuracy, given the strong (111) texture particularly in the Ru‐rich film. These results show that lattice contraction upon Ru addition is coupled with compositional complexity, where substantial atomic size mismatch – particularly between Ru and Au – generates lattice strain that either accumulates or relaxes locally.

**FIGURE 2:**
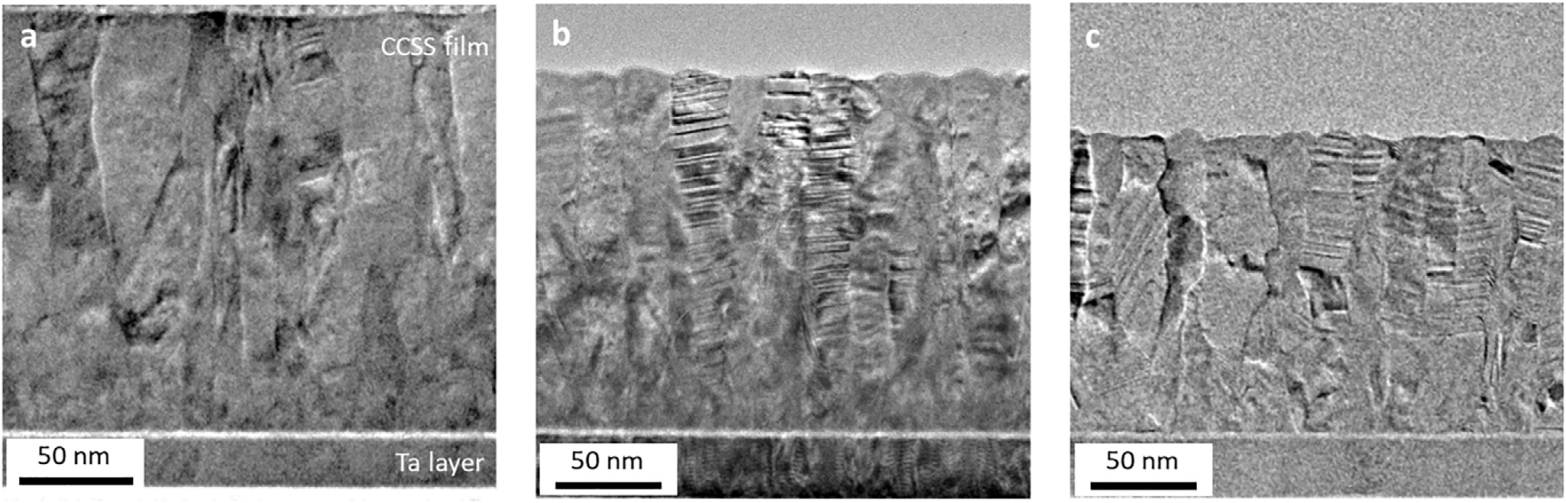
Grain morphology of three representative Au‐Pd‐Pt‐Ru CCSS thin films acquired in cross‐sections using TEM images (a) the Ru‐poor, (b) the equiatomic, and (c) the Ru‐rich thin films.

Cross‐sectional TEM bright field images (Figure 2) reveal columnar grains with film thicknesses of ∼145–185 nm. A Ta adhesion layer of ∼25–30 nm is observed below the films, enhancing bonding of the CCSS to the sapphire substrate. The reduction in film thickness with increasing Ru content originates from Ru's lower sputter yield compared to Au, Pd and Pt, leading to a reduced net deposition rate at the Ru‐rich area in the library [32]. Average grain sizes decrease with increasing Ru content: ∼30.2 ± 3.3 nm (Ru‐poor), ∼22.7 ± 2.0 nm (equiatomic), and ∼16.2 ± 2.2 nm (Ru‐rich). Planar defects which relax the lattice strain are visible in all three films. These planar defects are largely parallel to the substrate, but the contrast and number density differ, suggesting distinct defect morphologies across compositions.

**FIGURE 3:**
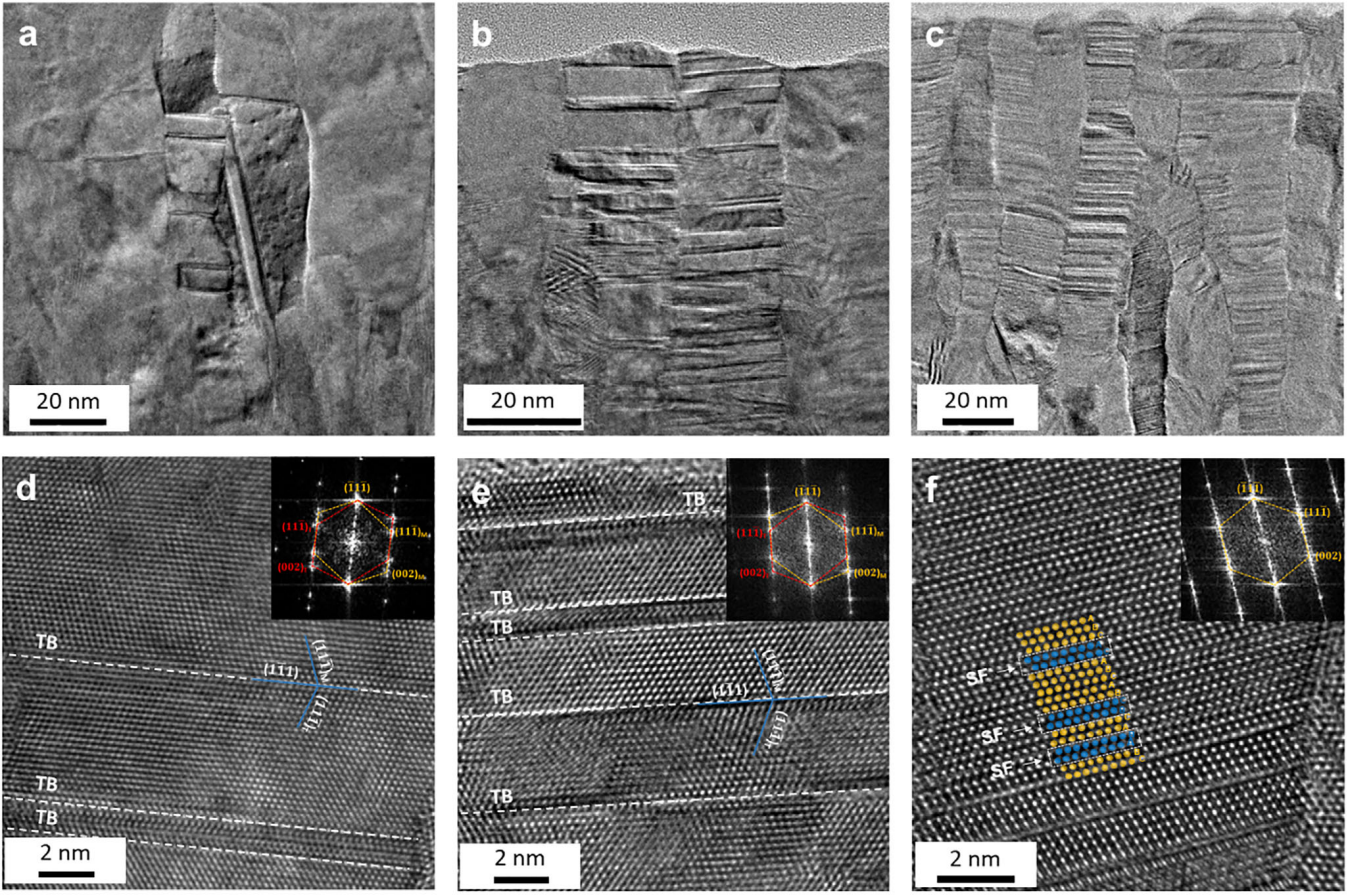
Planar defect formation of (a, d) the Ru‐poor, (b, e) the equiatomic, and (c, f) the Ru‐rich thin films. (d–f) are magnified HRTEM images, the insets show FFT patterns of twins (d,e) and matrix, indicated by orange and red lines, respectively.

To investigate these planar defects in detail, high‐magnification TEM images were obtained (Figure 3a–c). The Ru‐poor film contains relatively few planar defects which are separated by 7–20 nm, mostly aligned parallel to the substrate, with occasional oblique orientations. The equiatomic film exhibits a higher density of planar defects (1–10 nm wide), oriented predominantly perpendicular to the growth direction. In contrast, the Ru‐rich film shows dense arrays of planar defects with such small spacing that individual boundaries are difficult to resolve, again oriented largely perpendicular to the growth direction.

High‐resolution TEM (HR‐TEM) images (Figure 3d–f) further elucidate the nature of these defects. In the Ru‐poor film (Figure 3d), the fast Fourier transformation (FFT) patterns acquired along the [001] zone axis are indicated by dashed orange lines for the matrix and dashed red lines for the twin, showing mirror symmetry across the twin boundary (TB) and identifying the defects as nanotwins. The TB is marked by white dashed lines in the TEM image, and the annotated lattice orientations of the matrix, twin region, and the TB correspond to the indexing in the FFT patterns. In the equiatomic film (Figure 3e), similar FFT patterns confirm that the planar defects are also nanotwins; however, at the same magnification a higher density and smaller spacing between TBs are evident compared with Figure 3d. These twins are well aligned parallel to the substrate and exhibit growth along the {111} planes, consistent with the close‐packed orientation of the FCC lattice.

In contrast, the Ru‐rich film (Figure 3f) shows extensive SFs rather than nanotwins. The FFT pattern along the [011] zone axis displays streaking in addition to the diffraction spots, characteristic of SFs. To visualize the local stacking sequence, atomic columns in the matrix and within SF regions are marked by orange and blue solid circles, respectively; the accompanying sequence labels illustrate that the atomic sequence of FCC with ABCABC… order is locally disrupted by missing one {111} layer (e.g., ABCA(B)CAB…), which is a characteristic of an intrinsic SF. The high SF density indicates a local tendency toward HCP‐like ordering (ABABAB…), in line with Ru's thermodynamic preference for the HCP structure. As the Ru content increases, planar defect formation becomes more dominant, enabling partial structural relaxation toward HCP‐like stacking.

**FIGURE 4:**
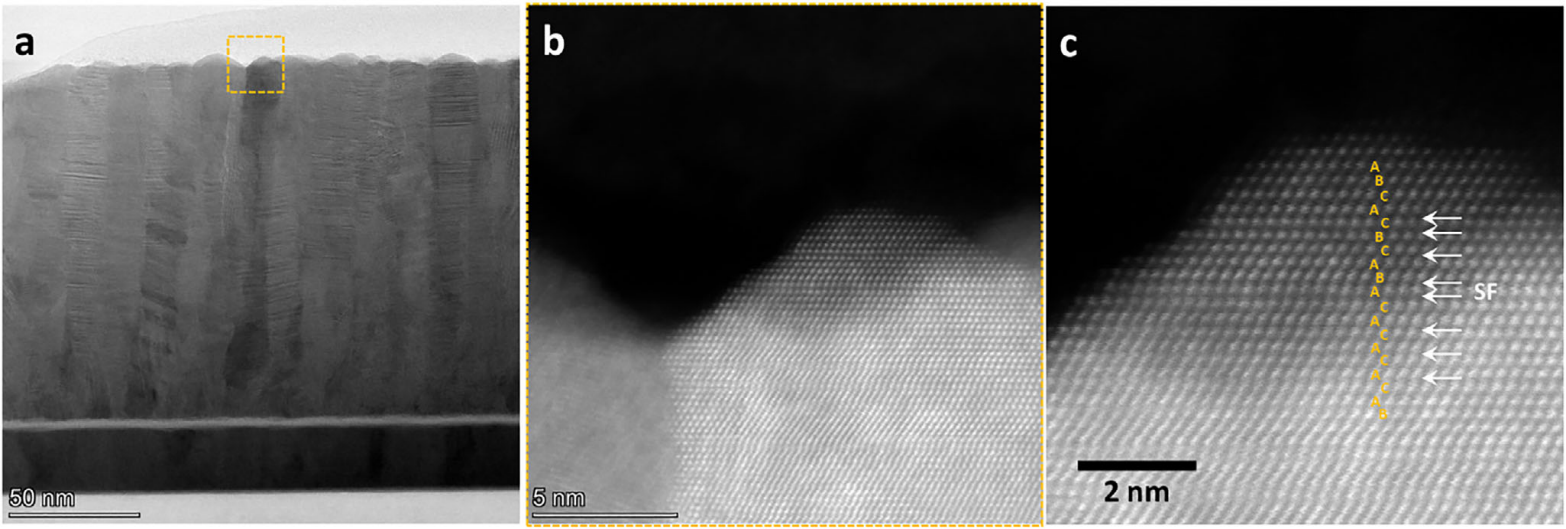
STEM images of the Ru‐rich film containing numerous SFs (a) low‐magnification STEM image of Ru‐rich, and (b) facet‐like surface structures of Ru‐rich. (c) shows SFs (marked by arrows; atomic stacking is labeled) in the surface layers.

For an ideally flat surface, such planar defects – if parallel to the surface – would indeed not influence the activity. However, the STEM observations reveal the surfaces of the films are rough (Figure 2). As shown for the Ru‐rich film (Figure 4), the facets form in the order of about 5 nm. For this representative area, approximately 20 atomic layers are terminating at the surface. Out of these 20 atomic layers, eight are SFs with different surface sites. Thus, they can strongly affect electrocatalytic activity.

**FIGURE 5:**
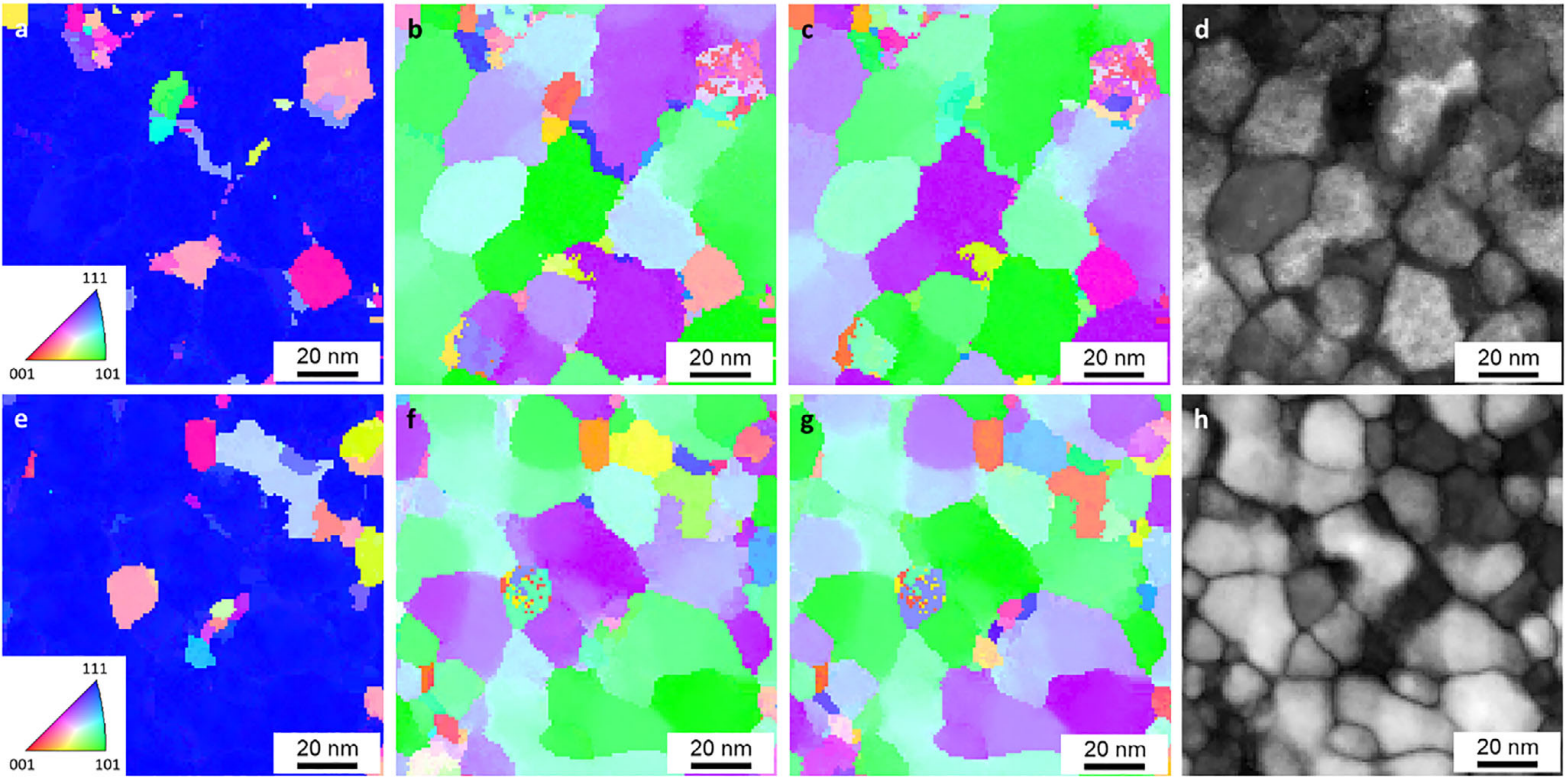
4D‐STEM orientation maps of plan‐view samples of (a–d) the equiatomic and (e–h) the Ru‐rich sample. (a, e) out‐of‐plane orientation maps, (b, f) in‐plane (100) orientation maps, (c, g) in‐plane (010) orientation maps, and (d, h) image quality maps of the equiatomic and the Ru‐rich thin films.

With alloying more Ru in the CCSS film, it is also necessary to investigate possible crystallographic orientation changes in addition to the planar defect formation. The orientation maps (Figure 5) acquired on plan‐view samples using four‐dimensional STEM (4D‐STEM) with a pixelated detector show out‐of‐plane (Figure 5a,e) and in‐plane (Figure 5b,c,f,g) orientations of the equiatomic and the Ru‐rich films. The 4D‐STEM analysis for the Ru‐poor film was skipped as the others showed a much higher electrochemical activity. The out‐of‐plane orientation maps of both thin films reveal a strong (111) texture, described by blue colors according to the legend, consistent with the XRD results. The strong (111) orientations reflect the low surface energy of FCC {111} planes, favoring growth along this direction during film deposition. However, the in‐plane orientation maps with sample orientations of (001) and (010) show random orientation distributions of both films. The image quality maps (Figure 5d,h) highlight GBs. The grain sizes are consistent with cross‐sectional TEM images.

**FIGURE 6:**
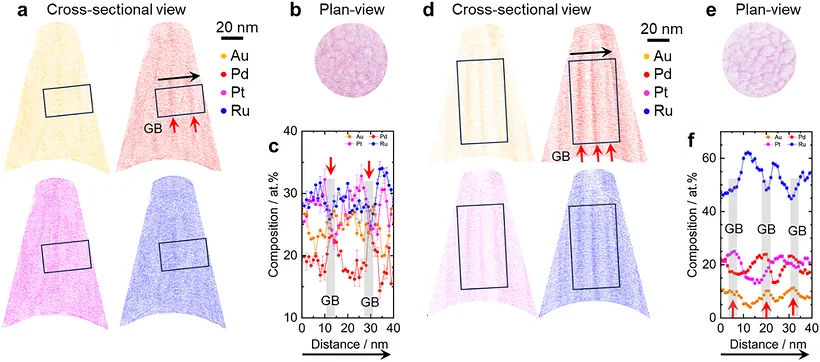
Elemental distributions of Au, Pd, Pt, and Ru in (a) the equiatomic and (d) the Ru‐rich viewed from the cross‐section of the APT needle‐shaped sample. Plan‐view of the atom maps for (b) the equiatomic and (e) the Ru‐rich. One‐dimensional (1D) composition profiles of (c) the equiatomic and (f) the Ru‐rich for the selected ROI shown in a, plotted along the black arrow direction indicated in the Pd map.

In addition to the grain interior, APT (Figure 6) was used to investigate the local atomic structure near defects, including GBs and planar defects in the equiatomic and the Ru‐rich films. Figure 6a,d reveal locally inhomogeneous elemental distribution, where Au, Pd, Pt, and Ru are marked in orange, red, magenta, and blue, respectively. Whereas the elements appear approximately homogeneous within grain interiors, chemical enrichment and depletion of specific elements at GBs is observed in both films, appearing much clearer in the Ru‐rich film than in the equiatomic film, as discussed in more detail below. Although local compositional fluctuations may form around TBs and SFs, these defects are not revealed in APT, which could be due to their high density, as shown by STEM (Figures 2 and 3). This leads to strong ion trajectory aberrations [33, 34] that degrade the spatial resolution of APT, causing smearing of the enrichment and resulting in element mixing around TBs and SFs. The absence of elemental enrichment at TBs and SFs could be due to the low energy of these defects, particularly when coherent TBs are present.

It is known that as‐deposited CCSS produced by sputtering exhibit a nanocrystalline structure with grains elongated along the film growth direction [35, 36]. A similar scenario is observed in the current alloys. Cross‐sectional images of our CCSS films (Figure 2) reveal the elongated grains in growth direction, whilethe plan‐view images show that the grains are equiaxed, with clearly visible GBs (Figure 5, and Figure 6b,e). Because the APT sample axis is parallel to the film growth direction and the spacings between the modulations match the grain sizes, the chemically modulated regions (Figure 6c,f) correspond to GBs. The black rectangle indicates the selected region of interest (ROI) across GBs for quantitative composition analysis. The red arrows mark the positions of two GBs. The grey bands in Figure 6c,f mark the corresponding positions of the GBs indicated in Figure 6a,d, respectively. Compositional analysis with magnified regions shows element‐specific enrichment and depletion at these boundaries. In the equiatomic and Ru‐rich films, Au and Pd are enriched at GBs relative to their contents in grain interiors, although the amounts differ. In the equiatomic film, line scans show depletion of Pt and Ru at GBs, whereas in the Ru‐rich film, depletion is observed predominantly for Ru. Notably, the Ru depletion at GBs is greater in the Ru‐rich than in the equiatomic film, indicating that the grain interiors of the Ru‐rich film contain more Ru than the nominal composition measured by SEM‐EDX. Similarly, the Au and Pd contents within the grains are lower than their nominal compositions in both films. In the region where the GBs terminate at the surface, different sites for the electrochemical reaction could evolve.

The observed enrichment (Au and Pd) and depletion (Ru) at GBs suggest that a higher content of Ru atoms in the grains is associated with SF formation. This is consistent with a thermodynamic preference of Ru to form the HCP phase. Au, Pd, and Pt have FCC structure but exhibit different stacking fault energies; Pd and Pt have higher stacking fault energies than Au [37], implying that higher Pd and Pt contents generally prevent the formation of twins and SFs, as twin formation energies scale with stacking fault energy. Given the high GB density in these CCSS films, GBs act as preferential segregation sites that drive local composition fluctuations in their vicinity. Furthermore, since the grain boundary energies of the constituents are larger than their stacking fault energies [38], excess atoms would preferentially be enriched at GBs rather than to the coherent interfaces of planar defects, and vice versa. These atomic configurations at defect structures could be governed by the formation energy of defects, which is also relevant to strain relaxation. The Ru atoms within the grains that are directly exposed to the (sub)surfaces could largely influence the electrocatalytic activities of the CCSS thin films.

As shown in Figure S1, the near‐surface composition was measured with XPS. The corresponding figure contains XPS data for all three films (Ru‐poor, equiatomic, and Ru‐rich) and they all exhibit a similar trend, with Pd enrichment and Pt depletion at the surface (Figure S2). The Ru content at the surface is comparable to that in the volume for all samples. Au is enriched at the surface of the Ru‐rich film and depleted at the surface of the Ru‐poor film, representing a relative compositional redistribution; nevertheless, the Ru‐poor (Au‐rich) film still exhibits the highest absolute Au content at the surface. This result reflects differences in surface energy among the constituent elements. In addition, Ru appears to be homogeneously distributed throughout the film, contributing more to lattice strain evolution and the planar defect formation rather than surface segregation.

In addition, the lattice strain arising from the mixing of multiple principal elements in the CCSS should be considered. The average lattice parameters of the three samples decrease as the Ru content increases in the Au‐Pd‐Pt‐Ru CCSS system, indicating lattice contraction in the Ru‐rich film. Furthermore, the further addition of Ru may induce SF formation in the Ru‐rich film, which can help to relieve accumulated lattice strain. However, lattice strain in the Ru‐rich film may still remain both at the surface and in the bulk. The lattice strain at the surface of the Ru‐rich film (Figure S2) was measured by geometric phase analysis (GPA) using STEM images [39]. GPA strain maps represent relative strain (relative displacement) with respect to a reference region. In particular, the strain map in the ε_*yy*_ direction shows a layered strain distribution, where compressive and tensile strain regions appear alternatively. For example, from top to bottom, compressive strain is observed in one strained region in the ε_*yy*_ direction, followed by tensile strain in the adjacent region, and this alternating pattern continues across the layers. These strain maps indicate that the strain is locally compensated rather than globally accumulated.

Therefore, the dominant measurable strain is likely defect‐associated, especially around SFs. The lattice strain arising from compositional complexity is real but may be too projection‐averaged, too weak, or too short‐ranged to be clearly resolved in conventional STEM strain mapping. However, this does not mean that lattice strain from compositional complexity does not exist. It is also not straightforward to disentangle the effects of SFs from those of lattice strain in order to determine which factor is dominant in enhancing electrocatalytic activity. Nevertheless, microstructural effects associated with planar defects – particularly when they terminate at surfaces – should not be underestimated when interpreting the enhanced electrocatalytic activity in CCSS.

In terms of active sites, Ru and Pt are efficient electrocatalysts for HER, and Pd is also known as an active electrocatalyst for HER. For example, even when Pt and Ru are well mixed in a binary alloy, they can contribute as an active site and directly influence the hydrogen binding energy. However, in the case of CCSS, the compositional complexity means that electrocatalytic activity is not only determined by the identity of the active element, but also by the neighboring atoms surrounding the binding site [17]. Even though Au is not widely regarded as a highly active element for the HER, it should not be considered completely inactive. HER activity on Au electrodes has been experimentally demonstrated [40], including under alkaline conditions, where the reaction kinetics can be strongly influenced by the electrode–electrolyte interface. In our high‐throughput dataset, selected MAs with an Au content of approximately 14 at.%, within the EDX uncertainty of ±1 at.%, require the potentials ranging from approximately −236 to −106 mV vs. RHE to reach −3 mA cm^−^^2^ as the Pt, Pd, and Ru concentrations change (Figure S3, Table S2 and Note S1). This observation demonstrates that the Au content alone cannot account for the observed variation in HER activity. Nevertheless, compared with Pt and Ru, Au is not expected to serve as the dominant highly active sites for the HER in the investigated Au–Pd–Pt–Ru CCSS. Instead, its role may be twofold: Au‐centered local environments may contribute comparatively less to the overall HER activity, while Au atoms in the coordination environment of Pt‐, Ru‐, or Pd‐centered sites can still modify their hydrogen‐binding properties and, consequently, their catalytic activity.

In CCSS electrocatalysts for HER, the local atomic arrangement of Au, Pd, Pt, and Ru, can tune the hydrogen binding energy and generate a variety of surface sites with different catalytic activities. In particular, Ru tends to promote the formation of planar defects, such as nanotwins and SFs, depending on the Ru content. Since these planar defects terminate at the surface, they can influence the local lattice structure and the surface atom arrangements. While compositional effects dominate in CCSS electrocatalysts, microstructural complexity may still play a supporting role in the electrocatalytic activity as defects terminating at the surface might change the active sites. The role of individual planar defects – if terminating at the surfaces – will be a focus of our future work.

## 3 Conclusion

This study investigates the effect of Ru content on the planar defect formation and HER electrocatalytic activity of Au‐Pd‐Pt‐Ru CCSS thin films. To obtain detailed structural information, three representative thin films were selected from a thin‐film materials library which was investigated for hydrogen evolution in alkaline media. With increasing Ru content, the lattice parameters decrease, while the strong (111) texture remains. Whereas the three films across the library show a single solid solution phase, various defect structures including GBs and planar defects are observed in the CCSS thin films. The GBs give rise to local atomic fluctuations, altering compositional distributions at an atomic scale and consequently influencing electrochemical activity at these sites. Notably, high densities of nanotwins and SFs are formed in the equiatomic and the Ru‐rich film, respectively. These planar defects exhibit coherent boundaries with the matrix, so enrichment at planar defects is scarcely observed. In contrast, specific elements are enriched or depleted at GBs owing to differences in the formation energies of GBs and planar defects. In particular, Ru is depleted at GBs in both the equiatomic and Ru‐rich films and tends to remain dissolved in the volume of the film rather than segregating at surfaces, indicating a higher Ru content within the grains and contributing to lattice strain. The films show a faceted surface on the nanoscale. In particular, the Ru‐rich film, containing a high density of SFs, shows that many of them extend to the surface and may modify its atomic structure. As a result, planar defects terminating at surfaces can influence electrocatalytic activity. This study provides guidance for the rational design of CCSS thin film electrocatalysts by considering both compositional and microstructural complexity.

## 4 Experimental Section

### 4.1 Synthesis of Au‐Pd‐Pt‐Ru CCSS Thin‐Film Materials Library

The Au‐Pd‐Pt‐Ru thin‐film materials library (ML) was synthesized using a four‐cathode magnetron co‐sputter system (Polaris, AJA International) using four 3.81 cm (1.5 inch) targets simultaneously (Au (99.99%, Sindlhauser Materials), Pd (99.9%, Sindlhauser Materials), Pt (99.99%, ESG Edelmetallservice), and Ru (99.99%, Evochem advanced Materials). A Ta (99.95%, Sindlhauser Materials) adhesion layer was sputtered onto a 10 cm diameter single‐side polished sapphire substrate (SITUS Technicals, c‐plane orientation) and moved under atmosphere condition to the co‐sputter system. All targets were pre‐cleaned for 120 s at 1.33 Pa and 20 W for DC and 30 W for RF power supplied targets. The deposition was carried out at room temperature with a deposition pressure of 0.5 Pa (base pressure 9.7 × 10^−5^ Pa) in Ar (99.9999%) atmosphere. All cathodes are equally distributed around the static substrate with a target‐to‐substrate distance of 10 cm. The positioning of each deposition source with respect to the substrate surface leads to wedge‐shaped deposition profiles. The overlay of these profiles from the four sources forms the continuous composition gradients of the ML. Based on deposition rate the deposition time was chosen to be 900 s to get an estimated layer thickness of 150 nm at the center of the ML. All other sputter parameters are presented in Table 1.

**TABLE 1:** Specific sputter parameter for the synthesis of the ML.

Targets	Au	Pd	Pt	Ru
Power Supply	RF	DC	DC	DC
Deposition Power (W)	30	16	16	29
Deposition rate (nm/s)	0.00576	0.00333	0.00362	0.00174

### 4.2 High‐Throughput Approach (Screening)

The ML was investigated using a high‐throughput approach for structural, compositional, and functional characterization. The chemical compositions of the ML were characterized automatically by measuring 342 measurement areas (MAs) (4.5 mm x 4.5 mm) using energy‐dispersive X‐ray spectroscopy (EDX) in a tungsten sourced scanning electron microscope (JSM‐5800 LV, JEOL) equipped with an EDX detector (INCA X‐act, Oxford Instruments) with a nominal measurement accuracy of ±1 at%. Before the characterization of the whole ML, a calibration on a Co standard was performed. Each MA was acquired for 60 s with an acceleration voltage of 20 kV at a magnification of 600. The quantification process was executed using *Oxford Instruments' INCA* software, yielding normalized compositions for each MA. Following the exclusion of the sapphire substrate signal, the analysis indicated that the ML center exhibited a composition of Au_30_Pd_28_Pt_19_Ru_23_.

The crystal structures of the ML were examined by X‐ray diffraction (XRD) using a diffractometer (D8 Discover, Bruker) equipped with a Cu X‐ray source (Incoatec IµS High‐Brilliance Microfocus, 0.15418 nm) and a 2D detector (Vantec‐500). Each MA 1D diffraction pattern was obtained by converting three merged 2D diffraction frames measured at 2θ positions of 30°, 50°, and 70°, covering a 2θ range of 20°–90°. Conversion and background subtraction were performed using Bruker's *DIFFRAC.EVA* software.

### 4.3 Electrochemical Measurements

All electrochemical measurements were conducted using a high‐throughput SDC setup, which automatically evaluates 342 MAs of the thin‐film materials library. The measurements were conducted in a conventional three‐electrode configuration using a Pt wire as counter electrode and Ag|AgCl|3m KCl reference electrode. The counter and the reference electrodes were placed inside the SDC tip made of polytetrafluoroethylene (PTFE) and filled with 0.05 m KOH electrolyte. Before each new measurement starts, the electrolyte is automatically replaced. By pressing the tip on the surface of the sample, an electrochemical cell is formed between the investigated MA (working electrode) and the electrolyte droplet. The circular area of the working electrode (0.00735 cm^2^) is defined by the tip opening (1 mm). The applied pressing force is monitored with a force sensor integrated into the tip holder. The measured current is normalized by the geometric surface area. All potentials are calculated and reported versus the RHE through the equation below where E_(Ag|AgCl|3M KCl)_ is the potential measured versus Ag|AgCl|3M KCl; 0.210 V is the standard potential of the Ag|AgCl|3M KCl reference electrode at 298 K; 0.059 is the result of (*RT*)/(*nF*), where R is the gas constant, *T* the temperature (298 K), *F* the Faraday constant, and *n* the number of transferred electrons during the reaction. The Nernst equation was used to calculate the values:

$$E_{\mathrm{RHE}}=E_{\left(\mathrm{Ag}|\mathrm{AgCl}|3M\;\mathrm{KCl}\right)}+0.210+0.059\;\mathrm{pH}$$

### 4.4 High‐Resolution (Scanning) Transmission Electron Microscopy

The HR‐TEM was carried out using an image Cs‐corrected Titan Themis 80—300 from Thermo Fisher operated at 300 kV, while high angle annular dark field – scanning transmission electron microscopy (HAADF‐STEM) with EDX analysis was conducted using a Thermo Fisher Titan Themis microscope with probe Cs correction at an accelerating voltage of 300 kV. STEM–EDX data were acquired using a Super‐X G1 system with four detectors at a tilted geometry. The quantified compositions were normalized to 100%. Signals from C, O, Cu, and Ga were excluded from the quantification. The 4D‐STEM was utilized for acquiring grain orientation maps. The data was collected using the electron microscope pixel array detector (EMPAD) equipped with the Cs‐probe corrected Titan Themis microscope. For the acquisition, a camera length of 1.5 m and a probe convergence semiangle of 0.65 mrad were employed. The 4D‐STEM data were reconstructed using the commercial software (automated crystal orientation mapping (ACOM); Nanomegas) with an input of crystal structure information of specimens. Cross‐sectioned and plan‐view TEM samples were prepared using a focused ion beam (FIB) system (FEI Helios G4 CX) operated at 30 kV. Low energy cleaning was also applied to each side of the TEM sample in the final step to remove beam damage. The cross‐sectioned TEM lamellae were used for investigating overview of microstructure in thin films, while the plan‐view TEM samples were used for acquiring the 4D‐STEM dataset for the orientation mapping. The Digital Micrograph software (DM Gatan) with a freely available GPA plugin [41] was used to measure the lattice strain from the STEM image of the Ru‐rich film.

### 4.5 Atom Probe Tomography Reconstruction

APT specimens were extracted parallel to the film growth direction using the same FIB (FEI Helios G4 CX), as mentioned above for TEM specimen preparation. Protection layer of about 20 nm thick carbon followed by a 200 nm thick Pt was deposited using an e‐beam on the selected area. APT analysis was performed using laser pulses on a local electrode atom probe (LEAP 5000XR, Cameca Instruments) at 65 K with 50 pJ laser energy at a pulse repetition rate of 125 kHz and a detection rate of 0.005 atoms per pulse. The APT data were reconstructed and analyzed using the AP suite 6.3 software.

## Author Contributions

**Miran Joo**: conceptualization, data curation, funding acquisition, methodology, project administration, resources, supervision, validation, visualization, writing – original draft, writing – review and editing. **Huixin Xiu**: conceptualization, data curation, formal analysis, investigation, methodology, validation, writing – original draft, writing – review and editing. **Sabrina Baha**: data curation, formal analysis, methodology, investigation, resources, validation, visualization, writing – original draft, writing – review and editing. **Ridha Zerdoumi**: data curation, formal analysis, methodology, investigation, resources, validation, visualization, writing – original draft, writing – review and editing. **Ningyan Cheng**: investigation, writing – review and editing. **Christoph Somsen**: formal analysis, funding acquisition, investigation, project administration, validation, writing – review and editing, writing – original draft. **Yujiao Li**: data curation, formal analysis, funding acquisition, investigation, methodology, project administration, validation, visualization, writing – original draft, writing – review and editing. **Aleksander Kostka**: data curation, investigation, methodology, Writing – review and editing, validation. **Wolfgang Schuhmann**: data curation, funding acquisition, project administration, validation, writing – review and editing. **Alfred Ludwig**: conceptualization, data curation, funding acquisition, project administration, resources, supervision, validation, writing – original draft, writing – review and editing. **Christina Scheu**: conceptualization, data curation, funding acquisition, methodology, project administration, resources, supervision, validation, visualization, writing – original draft, writing – review and editing.

## Funding

The authors acknowledge the Deutsche Forschungsgemeinschaft (DFG, German Research Foundation) in the – SFB 1625, project number 506711657, subproject B03 (M. Joo, H. Xiu, C, Somsen, and C. Scheu), subproject Z and A01 (S. Baha and A. Ludwig), subproject B01 (Y. Li), subproject C01 (R. Zerdoumi and W. Schuhmann), and S (A. Kostka). We thank Ibrahim Akinci for fabrication of the materials library as part of his student project.

## Conflicts of Interest

The authors declare no conflicts of interest.

## Supporting information




**Supporting File**: advs77600‐sup‐0001‐SuppMat.docx.

## Data Availability

The data that support the findings of this study are available from the corresponding author upon reasonable request.
