# Supplementary material for: Influence of Ru Content on Defect Formation and Electrocatalytic Activity of Au‐Pd‐Pt‐Ru Compositionally Complex Solid Solution Thin Films for the Hydrogen Evolution Reaction
Source: Adv Sci (Weinh). 2026 Sep 27:e77600. Online ahead of print. doi: 10.1002/advs.77600 (PMC13616268; doi:10.1002/advs.77600)
Supplement: Supplementary file 1 [file ADVS-9999-e77600-s001.docx]

Supporting Information

Influence of Ru content on defect formation and electrocatalytic activity of Au-Pd-Pt-Ru compositionally complex solid solution thin films for the hydrogen evolution reaction

Miran Joo^1^, Huixin Xiu^1,2^, Sabrina Baha^3^, Ridha Zerdoumi^3,4^, Ningyan Cheng^1^, Christoph Somsen^5^, Yujiao Li^6^, Aleksander Kostka^6^, Wolfgang Schuhmann^4^, Alfred Ludwig^3,6^, and Christina Scheu^1*^

Dr. Miran Joo, Prof. Dr. Huixin Xiu, Prof. Dr. Ningyan Cheng, Prof. Dr. Christina Scheu

^1^Nanoanalytics and Interfaces, Max Planck Institute for Sustainable Materials, 40237 Düsseldorf, Germany

E-mail: c.scheu@mpi-susmat.de

Prof. Dr. Huixin Xiu

^2^School of Materials and Chemistry, University of Shanghai for Science and Technology, 516 Jungong Road, Shanghai, 200093, People’s Republic of China

Sabrina Baha, Dr. Ridha Zerdoumi, Prof. Dr. Alfred Ludwig

^3^Chair for Materials Discovery and Interfaces, Institute for Materials, Ruhr University Bochum, 44801 Bochum, Germany

Dr. Ridha Zerdoumi, Prof. Dr. Wolfgang Schuhmann

^4^Analytical Chemistry-Center for Electrochemical Sciences (CES), Faculty of Chemistry and Biochemistry, Ruhr University Bochum, 44801 Bochum, Germany

Dr. Christoph Somsen

^5^Chair for Materials Science and Engineering, Institute for Materials, Ruhr University Bochum, 44801 Bochum, Germany

Dr. Yujiao Li, Dr. Aleksander Kostka, Prof. Dr. Alfred Ludwig

^6^Center for Interface-Dominated High Performance Materials (ZGH), Ruhr University Bochum, 44801 Bochum, Germany

E-mail: c.scheu@mpi-susmat.de

|  | **SEM-EDX** | | | **STEM-EDX** | | |
| --- | --- | --- | --- | --- | --- | --- |
| **at.%** | **Ru-poor** | **Equiatomic** | **Ru-rich** | **Ru-poor** | **Equiatomic** | **Ru-rich** |
| **Au** | 68 | 27 | 9 | 63 | 27 | 9 |
| **Pd** | 13 | 23 | 21 | 14 | 18 | 19 |
| **Pt** | 15 | 24 | 18 | 17 | 31 | 21 |
| **Ru** | 4 | 26 | 52 | 6 | 24 | 51 |

**Table S1.** Results of compositional analysis for the Ru-poor, the equiatomic, and the Ru-rich thin films, using SEM-EDX and STEM-EDX techniques.

**Figure S1.** Comparison of atomic concentrations of a) Au, b) Pd, c) Pt, and d) Ru in the bulk and at the surface of the Au–Pd–Pt–Ru CCSS material library, obtained by SEM–EDX and XPS, respectively. The linear fit to the scattered data is shown by the red dotted line in each graph, while the atomic fractions of the three films (Ru-poor, equiatomic, and Ru-rich) are highlighted. The black dotted line (slope ≈ 1) represents the case of no surface segregation.

Figure S2. a) STEM-HAADF image of surfaces in Ru-rich film, b)-d). Corresponding strain maps along $\boldsymbol{\varepsilon}_{\boldsymbol{xx}}$, $\boldsymbol{\varepsilon}_{\boldsymbol{yy}}$, and $\boldsymbol{\varepsilon}_{\boldsymbol{xy}}$ by STEM using GPA algorithm. e). The overlayed image HAADF image with $\boldsymbol{\varepsilon}_{\boldsymbol{yy}}$.

**Figure S3.** Spatial distribution and HER polarization behavior of selected MAs with an approximately constant Au content of 14 at.%. (a) Position of the selected MAs within the thin-film materials library. The highlighted areas correspond to MAs 94, 114, 155, 237, 292, 320, and 339. (b) LSVs of the selected MAs.

| **MAs** | **Composition (at.%)** | | | | **Potential vs. RHE (mV)** |
| --- | --- | --- | --- | --- | --- |
|  | **Au** | **Pd** | **Pt** | **Ru** |  |
| 94 | 14 | 49 | 7 | 30 | -236 |
| 114 | 14 | 45 | 8 | 33 | -172 |
| 155 | 14 | 39 | 11 | 36 | -155 |
| 237 | 14 | 26 | 19 | 41 | -134 |
| 292 | 14 | 18 | 26 | 42 | -112 |
| 320 | 14 | 13 | 33 | 40 | -106 |
| 339 | 14 | 9 | 42 | 35 | -135 |

Table S2. Selected composition with an approximately constant Au content of 14 at.% are arranged according to increasing Ru content and their HER activity. The potential required to reach a geometric current density of −3 mA cm⁻² was obtained from the corresponding LSV curves.

Supplementary Note 1. Effect of surface atom arrangement (SAA) in Au-Pd-Pt-Ru CCSS on electrocatalytic activity toward the HER

In our CCSS system, the four constituent elements of the Au-Pd-Pt-Ru CCSS should not be considered equivalent HER-active sites. This point is important for clarifying the SAA concept. The identity of the atom directly involved in the adsorption process strongly affects the adsorption properties, while the surrounding atoms modify these properties through the local chemical environment. Consequently, Pt-, Pd-, Ru-, and Au-centered SAAs can exhibit distinctly different hydrogen-binding properties and therefore different HER activities.

Regarding Au specifically, Au should not be considered completely inactive toward HER. HER on Au electrodes has been experimentally demonstrated^[1]^, including under alkaline conditions, and its kinetics are strongly affected by the electrode–electrolyte interface. Nevertheless, compared with Pt and Ru, Au is not expected to constitute the dominant highly active HER center in the investigated Au-Pt–Pd–Ru CCSS. Its role may instead be twofold: Au-centered arrangements may contribute comparatively less to the overall HER activity, while Au atoms located in the local coordination environment of Pt-, Ru-, or Pd-centered sites can still modify their hydrogen-binding properties. We further agree that the decrease in Au content can contribute to the experimentally observed increase in HER activity. Indeed, the Au-rich region of our composition-spread library exhibits comparatively low HER activity. However, our high-throughput dataset also shows that the Au concentration alone is not sufficient to describe the activity distribution. The library contains 342 individually measurement areas spanning Au contents from 9 to 69 at.%. At an identical Au concentration of 14 at.%, for example, the potential required to reach −3 mA cm⁻² varies from approximately −236 to −110 mV vs. RHE as the Pt, Pd, and Ru concentrations change **(Figure S3 and Table S2)**. Thus, compositions containing the same amount of Au can differ in HER activity by more than 120 mV. This observation demonstrates that reducing Au cannot by itself account for the complete activity trend. In the quaternary composition space, a decrease in the Au fraction necessarily occurs together with changes in the fractions of Pt, Pd, and/or Ru and, consequently, with changes in the statistical population of the different SAA. Therefore, the present composition–activity relationship should not be interpreted as a simple dilution of an inactive Au component. Rather, the experimental results indicate that the elemental composition controls the population and local chemical environment of HER-active surface sites.

In designing the CCSS platform, we carefully considered both the structural requirements for forming a stable CCSS phase and the relevance of the constituent elements to HER activity. Among the noble-metal candidates considered in addition to Pt, Pd, and Ru, which can serve as active sites for the HER, elements capable of supporting a stable FCC CCSS structure were initially prioritized. Other elements, such as Ir, Rh, or Ag, could certainly define scientifically interesting alternative compositional spaces. However, Ag was not selected here because of its relatively strong tendency toward surface segregation when alloyed with other noble metals, which could hinder the formation of a uniformly mixed CCSS surface. In terms of mixing enthalpy, Rh and Ir were considered less favorable for maintaining a well-mixed FCC solid solution with Pd and Pt under the conditions used in this study. Therefore, the final elemental combination was selected to satisfy the two main requirements of this work: the formation of a stable, compositionally complex FCC solid solution platform and the inclusion of catalytically relevant elements for the HER. A systematic comparison of different four elements was outside the scope of the present study.

References

1	Goyal, A. & Koper, M. T. M. The Interrelated Effect of Cations and Electrolyte pH on the Hydrogen Evolution Reaction on Gold Electrodes in Alkaline Media. *Angew Chem Int Ed Engl* **60**, 13452-13462 (2021). <https://doi.org:10.1002/anie.202102803>
